# Effect of the Adhesive Strategy on Clinical Performance and Marginal Integrity of a Universal Adhesive in Non-Carious Cervical Lesions in a Randomized 36-Month Study

**DOI:** 10.3390/jcm12185776

**Published:** 2023-09-05

**Authors:** Rainer Haak, Gesa Stache, Hartmut Schneider, Matthias Häfer, Gerhard Schmalz, Ellen Schulz-Kornas

**Affiliations:** Department of Cariology, Endodontology and Periodontology, University of Leipzig, Liebigstraße 12, 04103 Leipzig, Germany

**Keywords:** application mode, universal adhesive, non-carious cervical lesion, randomized clinical trial, FDI criteria, quantitative margin analysis

## Abstract

The effectiveness of a universal adhesive applied in three application modes for the preparation of Class V composite restorations was evaluated both clinically and by quantitative marginal analysis (QMA) over 36 months. In 50 patients, three (*n* = 21) or four (*n* = 29) non-carious cervical lesions (NCCL) were restored with Venus^®^ Diamond Flow (Kulzer GmbH, Hanau, Germany). The adhesive iBond^®^ Universal (iBU, Kulzer, Germany) was used in self-etch (SE), etch-and-rinse (ER), or selective-enamel-etch mode (SEE). The etch-and-rinse adhesive OptiBond^TM^ FL served as a control (OFL, Kerr GmbH, Herzogenrath, Germany). The restorations were clinically assessed (FDI criteria) at 14 days (BL), 6, 12, 24, and 36 months. Additionally, QMA was conducted on all restorations of 11 randomly selected patients. FDI criteria and marginal gap and perfect margin were compared between and within groups and recalls using McNemar, Wilcoxon, or Mann–Whitney U-tests (α = 0.05). Starting with 12 months, cumulative failure rates were lower in iBU-SE (0.0%, *p* = 0.016) and iBU-ER groups (2.1%, *p* = 0.07) compared to OFL (16.7%). At two years, iBU-SEE also showed fewer failures (0.0% SEE vs. 34.6% OFL, *p* = 0.016), as did iBU-SE compared to iBU-ER after 36 months (2.2 and 19.6%, *p* = 0.039). From BL, the iBU-SEE group always had the fewest marginal gaps and the highest percentage of perfect margins. From BL, iBU-SEE (0%, *p* = 0.008) and iBU-ER (0.2%, *p* = 0.027) showed significantly fewer marginal gaps compared to OFL (2.5%) and more perfect margins were found with iBU-SEE starting at 6 months (*p* = 0.054). The SEE and ER modes ensured the most excellent marginal quality, with differences from the control appearing earlier with QMA than clinically. In restoring NCCls, iBU showed superior clinical performance over OFL, especially in modes SE and SEE.

## 1. Introduction

Universal adhesives (UAs) are the latest generation of bonding systems developed to simplify clinical procedures and reduce technique sensitivity [1]. Also referred to as “multi-mode” or “multi-purpose”, these adhesives are designed to achieve equally effective bond strengths in self-etch (SE), etch-and-rinse (ER), and selective-enamel-etch (SEE) conditioning modes, according to the manufacturers [2,3,4]. This enables the dentist to handle the adhesive technique well adapted to the clinical situation [5,6,7].

In addition to microretentive adhesion, a chemical bond to the tooth structure is achieved by incorporating functional monomers [3]. Most UAs contain the functional monomer 10-MDP (methacryloxydecyl dihydrogen phosphate), which has a higher etching and more robust bonding capacity than other functional monomers [7]. According to the “modified adhesion route” described by van Meerbeck et al. [2,8,9], 10-MDP forms a stable ionic bond to calcium ions of hydroxyapatite and self-assembles in the hybrid layer into nano-layered calcium-monomer salts.

Numerous in vitro studies assessing and evaluating UAs have already been conducted. For enamel, additional phosphoric acid etching increased bond strength obtained in the laboratory [10]. In some studies, adequate microtensile bond strength (µTBS) to dentin was achieved regardless of whether ER or SE mode was used [3,9,11]. However, it has also been shown that in vitro bond strength, particularly on dentin, varies between different UAs [12]. Given that in vitro data cannot be uncritically extrapolated to the in vivo situation [13], simplifying clinical procedures does not always lead to the best clinical outcomes [14], and as manufacturers are constantly developing new products that appear to be even better, there is an ongoing need for clinical testing of new products.

To date, several clinical studies have been published on universal adhesives, reporting acceptable in vivo results [6,15,16,17,18,19,20,21,22,23,24,25,26]. SEE was the most recommended application mode for non-carious cervical lesions for better retention [27,28]. For iBond^®^ Universal (iBU), two randomized clinical trials have been published by other working groups [4,20,29]. Oz et al. [20] demonstrated acceptable clinical performance for iBU after 60 months of observation using a flowable resin composite. The retention rate was 92%, and for marginal adaptation, 65.2% and 34.8% of the restorations were rated Alpha and Bravo, respectively; for marginal discoloration, 73.9% and 26.1%; and for surface texture, 69.6% and 30.4%. All restorations were rated Alpha (100%) for color match, postoperative sensitivity, and secondary caries. However, only the SEE mode was used in this study, and no established reference adhesive was included for comparison. Thus, we started with a clinical study in non-carious cervical lesions, and iBU was evaluated in the restoration of non-carious cervical lesions being applied in all three conditioning modes: SE, SEE, and ER [29], with the additional use of the three-step etch-and-rinse adhesive OFL as a reference system. Clinical evaluation was conducted using FDI criteria [30,31].

Based on a previous study, the three key criteria “marginal staining”, “marginal adaptation”, and “fractures and retention” have been selected [15]. In order to evaluate the tooth–composite bond quantitatively and with high spatial resolution, in addition to the clinical evaluation in non-carious cervical lesions, the bond failure at the enamel–composite and the dentin/cement composite interface was investigated with optical coherence tomography (OCT). Already after the short observation time of 12 months with iBU, in general, lower cumulative failure rates were shown against OFL regardless of the conditioning mode. Consistent with this and based on OCT, already from BL, the universal adhesive showed fewer interfacial adhesive defects in all modes at the predominant dentin/cement composite interface. Additionally, more bond failures at the enamel–composite interface were seen in the SE mode from 6 months, compared to the SEE and ER modes and the reference system.

The present study extended the duration of the above clinical trial in non-carious cervical lesions by continuing the clinical evaluation for up to 36 months. In order to assess the marginal quality of the restorations, it was combined with quantitative marginal analysis (QMA). With this non-invasive method, the margins of restorations can be imaged and quantitatively assessed on replicas of restorations using scanning electron microscopy [32,33]. Based on the seminal work of Roulet et al. in 1989 [33], QMA is considered a proven method [34,35] to complement clinical evaluation criteria, which are less reliable when used alone.

Previous studies have shown QMA to be a predictive and sensitive method to assess tooth–composite interface integrity quantitatively and to evaluate adhesives [32,36,37,38,39,40,41,42]. Thus, this study determined the marginal gap and the perfect restoration margin. It is assumed that marginal integrity is defined by both the perfect margin and no marginal gap, both clinical and in QMA [37,38,42].

It was hypothesized that within the three-year study period:

(1) The universal adhesive would result in lower cumulative failure rates than the reference system in all application modes (*clinical performance of adhesives/application mode, primary outcome*).
(2) In QMA, the universal adhesive would show fewer marginal gaps and more perfect restoration margins than the reference system in all conditioning modes (*marginal quality, secondary outcome*).
(3) With time, marginal gap progression and a decrease in perfect margin can be detected (*gap progression*).
(4) Clinical restoration assessment and QMA are consistent in their statements. Group differences can be identified earlier by QMA (*method performance/power, tertiary outcome*).

## 2. Materials and Methods

### 2.1. Study Design (According to CONSORT)

The local Ethics Committee approved the randomized controlled clinical trial with reference number 294-15-13072015. It was registered at the German Clinical Trials Register (DRKS) DRKS00011064 (http://apps.who.int/trialsearch, accessed on 30 July 2015) and described in detail earlier [29]. The study was conducted from 2015 to 2019 (first clinical restoration 15 March 2016, last follow up 2 August 2019) following a four-arm parallel-group design, with each patient receiving at least three restorations from the four groups (randomized allocation). The patients, the investigators (clinical assessment (M.H.) and QMA (G.St.)), and the data evaluator (G.St.) were blinded about group affiliation. The adult participants were recruited at the Department of Cariology, Endodontology, and Periodontology of the University of Leipzig. They were informed verbally and in writing about the study and signed a declaration of consent. The treatment was performed by a dentist (G.S.) who was previously calibrated by placing 12 restorations in non-carious cervical lesions in vitro. In order to achieve the required quality and avoid composite excess during filling placement, the restorations were evaluated by OCT during calibration, particularly with regard to their marginal integrity.

### 2.2. Study Population

Fifty patients (mean age: 63.6 ± 12.8 years; 56% female and 44% male) with three (*n* = 21) or four (*n* = 29) non-carious cervical lesions (NCCLs) were enrolled. The minimum inclusion criteria for *n* NCCLs were three NCCLs in one patient. Thus, initially iBU was used in two NCCLs in the two modes SE and ER, and OFL was used in a third NCCL in ER mode; only if a there was a fourth NCCL was iBU applied in SEE mode. Individual age is given as age at the start of the study. A total of 179 teeth with a non-carious cervical defect were selected for restoration. Patients were included in the study if they were at least 18 years old and had a complete dentition with a minimum of 20 teeth without any removable dentures. After the start of the study, no changes were made to the inclusion and exclusion criteria for the patients or any of the methodological aspects, such as restoration procedures and restoration assessment. For the detailed description, see Merle et al. [29]. Each participant received composite restorations on three (21 of 50 patients) or four (29 of 50 patients, Table 1) non-carious cervical lesions on premolars, canines, and incisors. Lesions were allocated equally to an intervention group by a randomized computer-generated assignment performed by an independent member of the dental clinic not further involved in the study. The selection for QMA was performed after the 36-month follow-up examination. Only participants with continuous reassessment up to this point were included in the QMA analysis. On this condition, 11 of the 29 patients who received treatment for four NCCLs were selected using a four-block randomization. Furthermore, all restoration losses from patients who attended continuously over the 36-month study period were included in the QMA (23 of a total of 27 restoration losses). An overview of the selected restorations and their characteristics is given in Table 1. The sample size calculation was based on a pilot study by Schneider et al. [32]. These authors found significant differences between two study groups in a clinical restoration evaluation performed on 19 patients and by QMA when nine restorations (replica pairs) were included. Based on the group differences for the parameter marginal gap, it was calculated that a restoration pair number of *n* = 10 is required for QMA to achieve a test power of 80% (α = 0.05; PS-Power and Sample Size Calculation, version 3.0.43, Vanderbilt Univ., Nashville, TN, USA). In the present study, sample sizes of *n* = 50 and 29 for the clinical assessment and *n* = 11 for the QMA were chosen to ensure sufficient test power. At the study’s end, 24 participants with four non-carious cervical lesions each were available, 45 to 33 participants with three NCCLs, and 11 to 8 participants for QMA (Table 1).

### 2.3. Restorative Procedure

Each of the 179 cervical lesions received a restoration. For the detailed description, see Merle et al. [29]. Before shade selection, the trial teeth and surrounding tooth surfaces were cleaned with an oil- and a fluoride-free cleaning paste. As a rubber dam was not possible for most cervical lesions, a relative isolation was applied for contamination control. This included cotton rolls, retraction cords, and a four-hand-system for restoration placement. After placing a retraction cord (Ultrapak, Ultradent Products, Inc., South Jordan, UT, USA) and safeguarding permanent contamination control, the hypermineralized dentin and enamel margins were roughened using a 25 µm fine-grain diamond bur (Intensiv SA, Grancia, Switzerland). The adhesive iBond Universal (iBU, Kulzer GmbH, Hanau, Germany) was applied in the test groups in one of the three conditioning modes: SE, SEE, or ER. The etch-and-rinse adhesive OptiBond FL (OFL, Kerr GmbH, Herzogenrath, Germany) served as a reference standard. Patients with three lesions received two restorations using iBU in SE and ER application modes and a third restoration using the control system OFL. If a fourth lesion was selected, iBU was additionally applied in SEE mode.

Adhesive and composite (Venus^®^ Diamond Flow, Kulzer GmbH, Hanau, Germany) were applied according to the manufacturer’s instructions under contamination control (Table 2). Finishing and polishing were performed with fine-grain diamond burs (grain size: 15 µm) and rubber points (Shofu Dental GmbH, Ratingen, Germany). The entire restoration process was carried out using dental loupes (2.5× magnification).

### 2.4. Impression and Replica Production

After 14 days (baseline) and at each follow-up visit, impressions of the restorations were taken. Two impressions were taken with a low-viscosity A-silicone (Aquasil Ultra LV, Dentsply, Konstanz, Germany), whereby the first impression was discarded in each case in order to bind and remove any residual debris that might have remained despite surface cleaning. The study teeth were cleaned with a soft rotating brush and dried with oil-free compressed air. A small amount of impression material was blown into a thin film towards the sulcus and approximal region with a gentle air stream. Additional impression material was then applied until the impression achieved the required stability. The impression was removed, disinfected, and embedded in Aquasil Soft putty (Dentsply, Konstanz, Germany).

For replica fabrication conducted in the clinic’s research laboratory, the impressions were poured with an epoxy resin (Stycast 1266; Emerson & Cuming, Westerlo, Belgium) under controlled conditions. The replicas were trimmed, mounted with carbon (Leit-C-Plast, Neubauer Chemicals, Münster, Germany) on metal sample plates (sample plate with pen, 12 mm, Plano GmbH, Wetzlar, Germany), and sputtered with gold (10 nm, LOT MiniSputterCoater Automatic MSC1T, Liebscher GmbH, Schöffengrund, Germany). For a blinded evaluation, they were randomly labeled with a number later decoded for statistical analysis.

### 2.5. Study Outcomes

#### 2.5.1. Clinical Evaluation

**Figure 1:**
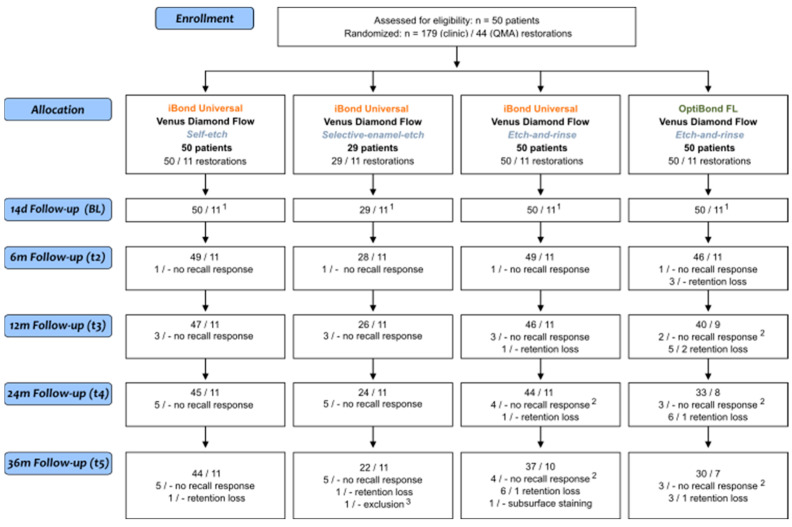
CONSORT flow diagram; ^1^ restorations in situ (BL to 36 months); ^2^ not including restorations lost before; ^3^ root canal treatment on the trial tooth.

The principal examiner (MH) performed clinical restoration evaluation and impression-taking at the same dental chair at each recall. All restorations were assessed after 14 days (baseline, t1), 6 months (t2), 12 months (t3), 24 months (t4), and 36 months (t5) (Figure 1) according to the FDI criteria [30,31]. The aesthetic, functional, and biological criteria were evaluated visually with dental magnifying glasses (2.5×), by using explorers (Kit-EX: tip diameter 150 µm, 250 µm; Deppeler SA, Rolle, Switzerland), by interviewing, by CO_2_ snow, by use of a visual analog scale, and by a periodontal probe (P15/11.5B6; Hu-Friedy Mfg. B.V., Rotterdam, The Netherlands).

The rating scores were 1 (very good), 2 (good, after correction very good), 3 (sufficient/satisfactory, minor shortcomings), 4 (unsatisfactory, but repairable), and 5 (poor, replacement necessary). If a restoration did not receive a score of 1 for marginal adaptation at baseline examination, minor marginal fractures were removed until they could be assessed with a score of 1.

Restorations were excluded from further examination if they were rated clinically unacceptable (clinical failure, score 4 or 5) in any criteria. If so, they were repaired or replaced. For documentation, the study teeth were photographed before and after restoration and at each follow-up visit. The criteria fractures and retention, marginal adaptation (MA), and marginal staining (MS) defined the clinical endpoints.

#### 2.5.2. Quantitative Margin Analysis

Scanning electron microscopy (Phenom G2, Phenom-World BV, Eindhoven, The Netherlands; 5 kV, 200× magnification) was used to image the restoration replicas by the investigator (GS). The images were stitched together (Fiji/ImageJ version 2.1.0/1.53i and plugin MosaicJ [43]), and the restoration margin criteria were assessed (Fiji/ImageJ version 2.1.0/1.53i and plugin QuantiGap [44]) by the investigator, who was calibrated by an experienced operator (MW, see [42]).

**Figure 2:**
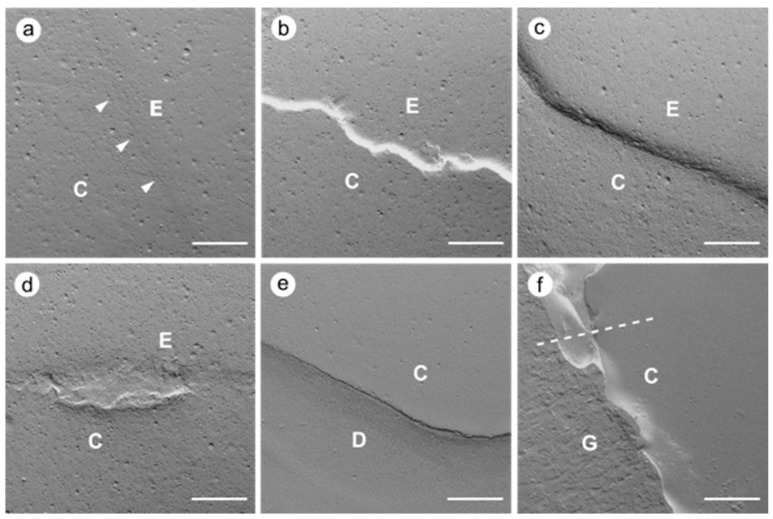
Exemplary cases of the six criteria used in the quantitative margin analysis (SEM images, 200× magnification); (**a**) perfect margin (white arrowheads), (**b**) positive ledge, (**c**) negative ledge, (**d**) margin irregularity, (**e**) marginal gap, (**f**) artifact, gingiva overlays the restoration margin which can only be assessed above the dashed line; C—composite, E—enamel, D—dentin, G—gingiva; scale: 200 µm.

**Figure 3:**
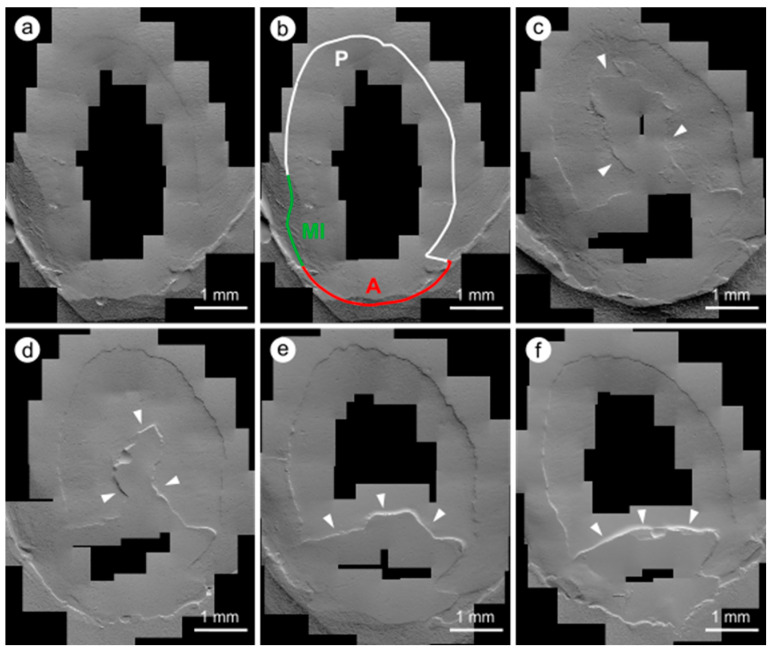
SEM images of a restoration at different examination time points over 36 months, tooth 13, iBU-ER due to fractures and chipping of the composite (white arrowheads), a decline/variation in the total length of the restoration margin can be observed; (**a**) baseline, (**b**) QMA at baseline (MI—margin irregularity, P—perfect margin, A—artifact), (**c**) 6 months, (**d**) 12 months, (**e**) 24 months, (**f**) 36 months.

**Figure 4:**
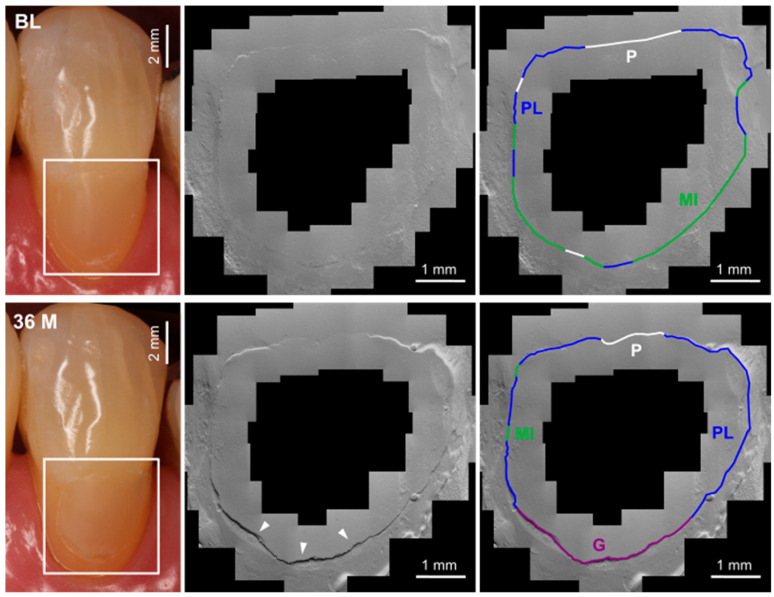
Clinical (**left**, restoration within the white frame) and SEM (**middle**) imaging and the margin characteristics (**right**) of the restored tooth 13, iBU-ER; BL (baseline) clinical evaluation: marginal staining score 1, marginal adaptation score 2 after minor corrections, fractures and retention score 1, QMA: no gap formation can be detected; 36 M (36 months) clinical evaluation: marginal staining score 1, marginal adaptation score 3, fractures and retention score 1, QMA: a marginal gap formation in the cervical region of the margin can be seen (white arrows heads, purple line); MI—margin irregularity, P—perfect margin, G—marginal gap, PL—positive ledge.

The following six evaluation criteria were used: perfect margin (PM), positive ledge (PL), negative ledge (NL), marginal gap (G), margin irregularity (MI), and artifact (A, impression artifacts such as deposits and bubbles as well as gingival overlays), whereby the artifact length was subtracted from the total length of the restoration margin (Figure 2, Figure 3 and Figure 4). The two criteria “gap” and “perfect margin” were considered following Haak et al. [41]. The percentages of “marginal gap” and “perfect margin” were determined in relation to the total restoration margin length (without artifact length) for each imaged restoration. For further statistical analysis, the arithmetic means for both criteria were calculated.

### 2.6. Statistical Analysis

SPSS software for Windows (version 27.0, IBM Corp. Armonk, NY, USA) was used to analyze clinical and QMA data statistically. A significance level of α = 0.05 was set, with *p*-values ranging from 0.05 to 0.07 estimated as a trend. Due to the exploratory nature of this study, *p*-values were reported as raw *p*-values, and no adjustment for multiple testing was made. For each group per follow-up assessment, the cumulative failure rate (CFR) for each criterion and the sum of all criteria were calculated using the following formula: Failure rate (%) = [(F_previous_ + F_current_):(F_previous_ + N_current_)] × 100%. F_previous_ represents the number of previous failures before the current examination, whereas F_current_ and N_current_ constitute the number of failures and the number of restorations seen in the current recall.

A clinical failure occurred when one of the clinical criteria was scored as 4 or 5. The McNemar test (two-sided) was performed to compare the results between study groups at each recall (horizontal testing) and within a group from baseline over time (longitudinal testing). For restorations that were lost (fractures/retention, score 5) or clinically unacceptable (marginal staining, score 5), the missing value in this group was replaced by a score of 5 at later times (missing data imputation) following Haak et al. [41]. For the target test power of 80% (α = 0.05), the randomized selection of eleven patients with four restorations who appeared consistently and had 23 of the total 27 restoration losses was performed for the QMA. Four lost restorations were not included as the patients did not appear continuously from BL to 36 months. The arithmetic mean values of the criteria “marginal gap” and “perfect margin” per group were determined, and the groups were statistically compared. If a restoration was lost, the missing value for “marginal gap” was replaced with the highest value measured at that time in the respective study group (missing data imputation). In case of missing values for the criterion “perfect margin”, they were substituted with the smallest value according to the same principle.

Data were tested for normal distribution by graphical plotting, Smirnov–Kolmogorov test, and Shapiro–Wilk test. Nonparametric tests were used for further analysis if the normal distribution assumption was violated. Friedmann and Wilcoxon tests were performed to compare the study groups (Table 1; randomized patient selection, *n* = 11) at each follow-up assessment (cross-sectional testing) and within a group between examination time points (longitudinal testing). The Mann–Whitney U-test was used to compare values of lost and remaining restorations. In the groups iBU-ER and OFL, the remaining fillings within the randomized selection (*n* = 11) were compared with the losses in the respective group. The test was not performed in the iBU-SE and iBU-SEE groups, as only one restoration was lost in each (Table 1). The interpersonal variance between the calibrated QMA investigator (GS) and two other anonymous raters experienced in QMA (3 years) was calculated on five randomly selected replicas. The standard errors of the means were ≤1.6% in the criterion “marginal gap” and ≤11.9% in the criterion “perfect margin”. Intrapersonal variance (GS) was determined by three measurements per replica. The standard errors of the means were ≤0.2% in the criterion “marginal gap” and ≤1.2% in the criterion “perfect margin”.

## 3. Results

### 3.1. Clinical Evaluation

At 36 months, 145 of the 179 restored teeth were examined, resulting in a reassessment rate of 66.0% to 90% in the respective groups. A detailed listing of the follow-up rates and the percentages of non-acceptable restorations (%) in the esthetic, biological, and functional criteria or the cumulative failure rate is shown in Table 3 and Figure 1. The percentages and group differences (p_i_) in the criteria “marginal staining” (MS, score 2 or 3), “marginal adaptation” (MA, score 2 or 3), “fractures and retention” (FR, score 5), and “cumulative failure rate” at each time (horizontal testing) and differences per group from BL to 36 months (longitudinal testing) are summarized in Table 4 and Table 5. A rating as clinically unacceptable (score 4 or 5) occurred in the two criteria “marginal staining” and “fractures and retention”. The OFL group, with 17 failures, had the highest number of retention losses: three after six months, five after 12 months, six after 24 months, and a further three after 36 months. These failures resulted in an increasing cumulative failure rate, from 6.1% at 6 months to 36.2% at 36 months (Table 3, Table 4 and Table 5, significant from 12 m). In the iBU-ER group, one restoration was partially or entirely lost at 12 and 24 months and a further six at 36 months. Because of subsurface staining, another restoration was excluded after 36 months. Starting with a CFR of 0% at baseline, a significant increase up to 19.6% after 36 months was observable in this group. In iBU-SE and iBU-SEE groups, one restoration each was lost at the 36-month examination with no significant increase in CFR within the period (Table 5). The highest failure rate in fractures and retention in the OFL group is significant compared to the iBU-SE group as early as 12 months and to all iBU groups at 24 and 36 months. Except for the comparison of iBU-ER vs. OFL at 36 months, the same applies by analogy to the CFR. Within the iBU groups, most restoration losses occurred in the iBU-ER group, with a significantly higher CFR at 36 months than in the iBU-SE group (2.2% < 19.6%, Table 4). All groups had a significant shift from score 1 to score 2 or 3 for MA and MS criteria from 6 months (MA, groups iBU-SE, iBU-ER, OFL) or 12 months (MS, all groups, Table 5). No significant differences were found between groups when comparing MA and MS criteria horizontally (Table 4). Only in group OFL was there a trend toward a higher percentage of score 2/3 in the MA criterion compared with group iBU-SE after 12 months.

### 3.2. Quantitative Margin Analysis

**Figure 5:**
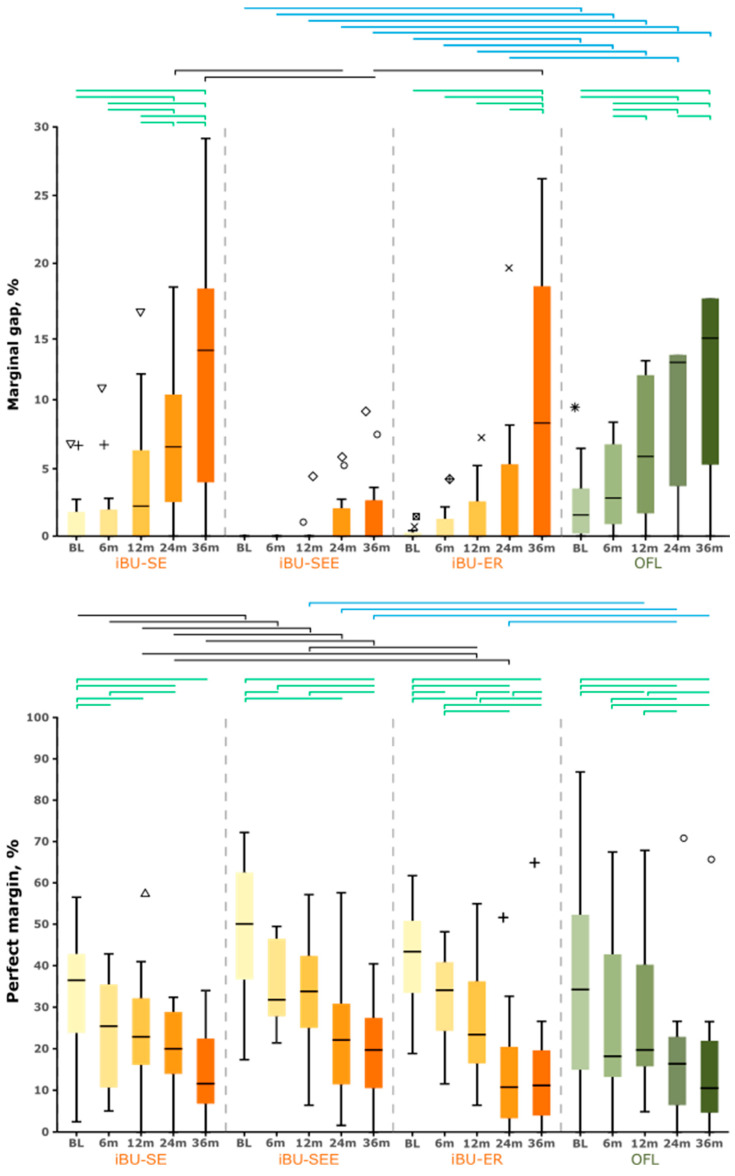
Boxplots of mean marginal gap (%) and perfect margin (%) of restorations in the groups iBond Universal and OFL for the 14-day (BL), 6-, 12-, 24-, and 36-month follow-ups. Significant group differences (*p*_i_ < 0.05) at each follow-up (black, blue) and significant gap increase/decrease in perfect margin in the groups over time (green) are marked. Identical symbols (triangle, circle, cross, asterisk) indicate recurring outliers of the same fillings.

The SEM examination results are shown in Table 6 and Table 7 and Figure 5. Marginal gap formation was found mainly in the cervical region of the restoration margin in all study groups. During the observation period, a change in margin quality was detected: the marginal gaps increased, whereas the proportion of perfect margins decreased over time. Marginal fractures were observed, which caused a shortening of the measured total restoration margin length (Figure 2, Figure 3 and Figure 4). The slightest changes occurred in group iBU-SEE. In particular, the marginal gap did not increase significantly in this group during the investigation period. In contrast, the iBU-SE and OFL groups showed a significant increase in the marginal gap from BL to 24 months and more frequently between the different examination time points, while in the iBU-ER group, a significant increase in marginal gap occurred only after BL to 36 months and less frequently between the recalls (longitudinal testing, Supplementary Table S1). In the criterion “marginal gap”, differences between individual study groups were already statistically verifiable after 14 days (cross-sectional testing, Table 6, Figure 5): the control group OFL showed significantly higher gap values compared to the groups iBU-SEE (*p* = 0.008) and iBU-ER (*p* = 0.027). This result was reproducible up to 36 (iBU-SEE) or 24 months (iBU-ER). Compared to the marginal gap, a significant decrease in perfect margins was observed in all groups and earlier than the increase in gap values. As early as t2 in three groups (iBU-SE, iBU-SEE, iBU-ER), a significant decrease in perfect margin was shown.

From 24 to 36 months, there was a sudden increase in marginal gap in the iBU-ER group reaching almost the same mean value as in the OFL group. At no time point was there a significant difference between the iBU-SE and OFL groups. In general, the lowest mean values for marginal gaps were observed in the iBU-SEE group through the whole observation period, and in particular, no marginal gap was even observed in the first six months (Figure 5). The difference with the iBU-SE group was significant at 24 months, and the difference with all other groups was significant at 36 months.

In contrast, in the iBU-SE group, the highest marginal gap mean values resulted within the iBU groups throughout the follow-up period (analogy: no significant differences compared to the control group). In the evaluation of the parameter “perfect margin” in the iBU-SEE group, significantly higher values compared to the iBU-SE group at all examination points were observed (p_i_ ≤ 0.032), and compared to the OFL group, this was observed from t3 to t5 (p_i_ ≤ 0.014). In contrast, isolated significant group differences were found in the comparisons iBU-SEE/iBU-ER at t3, iBU-SE/iBU-ER at t3/4, and iBU-ER/OFL at t4 (Table 6). Analogous to gap evaluation, no significant differences between the iBU-SE and the control group could be detected (p_i_ ≥ 0.175). No significant differences were found between lost and remaining fillings in the criteria marginal gap and perfect margin (Table 7). In addition, bent-up restoration margins were found more often in the microscopic images of the lost restorations (17.9% of remaining restorations vs. 39.1% of lost restorations).

### 3.3. Clinic and Quantitative Margin Analysis

During the 36-month observation period (longitudinal testing), both assessments showed a significant decrease in the quality of the tooth–composite bond in all groups, with noticeable differences between clinical evaluation and QMA. Clinically, there was a significant shift from score 1 to score 2/3 in the criterion MA from 6 months onward in the groups iBU-SE/ER and OFL and the criterion MS in all groups from 12 months onward. Fracture/retention and cumulative failure rate did not increase significantly in the iBU-SE and iBU-SEE groups. In contrast, there was an increase in the control group OFL from 12 months and in group iBU-ER after 36 months compared with BL (Table 5). QMA also resulted in significantly fewer perfect margins in all iBU groups from six months, while marginal gaps increased significantly from 24 months in the iBU-SE and OFL groups (Supplementary Table S1). From BL to 36 months, no significantly increased marginal gap was observed in the iBU-SEE group, in contrast to the clinically observed significantly decreased marginal adaptation from 24 to 36 months.

The comparison of the groups at the time of the examination (horizontal testing) also shows agreements and differences between the two assessments. The high retention loss observed in the control group OFL from 6 months onwards, which was significantly higher from 12 months compared to the iBU-SE group and from 24 months compared to all iBU groups, is in principle confirmed by the more extensive marginal gaps recorded in QMA in this group compared to the iBU groups. This was significant compared to the iBU-SEE group (BL to 36 m) and iBU-ER group (BL to 24 m). The difference to group iBU-SE, however, was not significant at any time (Table 6). The iBU-ER group had the second-highest retention loss. At the 36-month assessment, six restorations were lost, reflected in a substantial increase in the marginal gap from 24 to 36 months (3.7% to 11.6%) with no more significant difference to the control group concerning CFR.

Contrary to the clinic, where a significantly higher CFR was observed in the iBU-ER group than in the iBU-SE group at 36 months, fewer perfect margins were observed in the iBU-SE group compared with the iBU-ER group at 12 and 24 months. Even significantly fewer perfect margins were noted in the iBU-SE group at all examination time points compared with the iBU-SEE group. At 24 and 36 months, there were also significantly higher gap values than in the iBU-SEE group. No significant differences were found between MA and MS groups at clinical evaluation. The group differences (QMA) that can be represented with marginal gap and perfect margin are thus not reflected in the clinical criterion MA. When comparing QMA and clinical assessment, the parameter perfect margin was less sensitive and inconclusive.

## 4. Discussion

In this clinical study, iBU showed less retention loss than OFL at 36 months, regardless of the conditioning mode used. While the bonding strategy of the ER adhesive OFL is based on micromechanical anchorage, iBU can additionally establish a chemical bond to the tooth structure. It contains the functional phosphate monomer 10-MDP, which forms a robust ionic bond with the calcium ions of the hydroxyapatite [45]. OFL contains glycerophosphate dimethacrylate (GPDM) [46,47], which does not form stable salt compounds with the tooth substances [46,47]. In contrast, iBU appears to establish a more durable long-term bond with the tooth structure. This is reflected in a higher clinical performance, demonstrated statistically from 12 months for the self-etch mode and 24 months for all three application modes of iBU. Based on the results of the study, (Null-) hypothesis 1 (*clinical performance*) could not be rejected. The perceptible differences between the iBU-SEE and iBU-ER groups and the control group OFL could not be statistically verified after 12 months. On the one hand, most probably this was due to the smaller sample size with 29 instead of 50 restorations in the iBU-SEE group and, on the other hand, there was one restoration loss in the iBU-ER group after 12 months. Yet, fifty subjects with at least three lesions were scheduled for the study. The fact that patients with four NCCLs worthy of restoration are rarely available and that patient enrollment had to be completed within a limited time frame necessitated the lower sample size for the iBU-SEE group.

QMA confirmed most clinical outcomes: Restorations of iBU-SEE and iBU-ER groups had lower marginal gaps and higher proportions of perfect margins than the control group, which was already observed at BL and continued over the observation interval. While iBU-SEE showed increased marginal integrity compared to OFL at all time points, the marginal gaps in the iBU-ER group increased to the level of the control group at 36 months, such that the difference from OFL could no longer be verified at this time point, even for the clinical criterion CFR. In SE mode, however, no significant difference could be found in QMA with iBU compared to the three-step control adhesive. Hypothesis 2 (*marginal quality*) must therefore be partially rejected.

In a randomized clinical trial with iBU in NCCL restorations, Oz et al. [4] stated that at 18 months, a retention rate of 96.8% was achieved in SEE mode, and 90% of the restorations were rated Alpha (UNC-modified USPHS criteria) for marginal discoloration and marginal adaptation. After 60 months, acceptable clinical performance was still achieved, with a survival rate of 85.2%. There was no significant difference in retention compared to two other universal adhesives, both also applied in SEE mode [20]. Merle et al. [29] recently published the 12-month results of the present clinical study, in which iBU had a failure rate of 0% in SEE or SE mode and 2.1% in ER mode. At 36 months, with a CFR of 2.2% in SE mode and 4.3% in SEE mode, we continued to see acceptable failure rates and thus retention rates as high as Oz et al. found in SEE mode [6]. This was also true for iBU-ER up to 24 months but in contrast to the higher CFR in ER mode of 19.6% in the subsequent 24 to 36 months. In addition, the QMA indicates that the marginal gap and perfect margin in group iBU-ER were intermediate between the values of groups iBU-SEE/SE up to 24 months. In the etch-and-rinse mode, the deterioration of the interface with a longer dwell time could be caused by an initially weaker interaction with the dentin.

OFL is considered an established reference system, although it does not always perform best based on previous studies [48,49,50,51,52]. When used to restore NCCLs, it has shown in previous studies acceptable failure rates of 0% [48] and 4% [49] after one and 9% [53] after five years of observation. This contrasts the high failure rate of 36.2% after three years observed in this study. In another study by the authors examining Scotchbond Universal adhesive, OFL also showed a high failure rate of 20% after one year [15]. In this and the current study, the restorations were placed by one dentist each, but the dentist differed between the studies. Both were extensively calibrated and had many years of experience with OFL. One explanation of the phenomenon could be derived from the mechanical and chemical preparation of the cavities’ surfaces. In NCCLs, up to 10 µm of sclerotic dentin is present at the surface. This zone with denatured collagen and obliterated dentinal tubules does not provide optimal conditions for the micromechanical anchorage of an adhesive [54,55]. Some authors recommended dentin roughening for better retention [56,57,58]. It can be assumed that the mechanical roughening and etching of the cavity surface was not enough to remove the sclerotic dentin superficially, indicating that the collagen network and the dentinal tubules were not sufficiently exposed for penetration of the monomers [58]. Standardization for the mechanical preparation of the cavity is difficult to achieve. However, it is assumed that especially for OFL, a stronger roughening or more extended etching (>15 s) might be necessary to achieve a sufficient bond. iBU, conversely, is less dependent on micromechanical bonding due to the additional chemical bond explaining the enhanced clinical retention. This is in line with the findings of a randomized clinical trial, where universal adhesives in NCCLs did not have higher retention in roughened dentin compared to unprepared dentin, irrespective of the conditioning mode [59].

In general, an increase in marginal gaps and a decrease in perfect margins were observed over the 36 months, so hypothesis 3 (*gap progression*) can be accepted. The progression of the marginal gaps can be explained by the increasing degradation of the hybrid layer by enzymatic, chemical, and microbiological processes [11]. Mechanical stress due to masticatory loading further promotes marginal gap progression. The decrease in marginal quality seen in the SEM images is consistent with the clinically observed increase in marginal discoloration, the decrease in marginal adaptation, and the increasing loss of retention. Thus, bacteria, metabolites, and fluids can penetrate the marginal gap, which may promote these clinical observations [60]. Marginal gap progression was observed mainly in the iBU-SE and OFL groups, whereas gap values remained almost stable in the iBU-SEE group. Thus, the groups that already had poorer marginal integrity initially also showed a greater decrease in marginal quality over time. Within the observation interval, the gap values increased significantly only after two years, while a significant decline in perfect margins already occurred after six months. The progression and sequence of the parameter criteria can explain this: a “perfect margin” may first change to a “positive ledge” or “margin irregularity” even before a marginal gap becomes visible; not every criterion is chronologically mandatory, but they can also be skipped. Lost restorations did not show higher values for marginal gap or a less-than-perfect margin in the QMA prior to the loss. It was impossible to characterize a loss of restoration that had occurred at some point by an initial deficient marginal seal.

In order to assess the clinical success of a restoration and to identify differences between different materials or application modes, long-term clinical studies are required [41]. For the criteria “marginal staining” and “marginal adaptation”, all fillings in all groups were clinically acceptable at 36 months without significant group differences. In contrast, significant group differences were first observed clinically at 12 months by lower retention in OFL compared to the iBU-SE group. In QMA, on the other hand, significant group differences were already found at baseline. iBU-SEE and iBU-ER already had significantly more gap-free restoration margins than OFL at this time. Clinically, this was reflected in significantly less restoration loss only after a time delay of 24 months. Therefore, QMA has a higher discriminatory power, allowing early assessment of bond quality with fewer subjects in each group. It is a valuable tool for obtaining additional information, as minor marginal fractures and irregularities can be detected before they become clinically apparent. In general, we expected the clinical outcome to be related to the marginal quality of the restorations evaluated by QMA. Most of the results of the two evaluation methods confirmed each other, as the groups with less favorable clinical retention also had poorer marginal integrity, which is particularly evident for the iBU-ER group. In 24 to 36 months, the increase in retention loss and the decreasing marginal quality shown with QMA correspond to each other. Nevertheless, the better clinical performance of the universal adhesive in the SE mode compared to the control group could not be confirmed with QMA; there was no significant difference compared to OFL at any time. Therefore, hypothesis 4 (*method performance*) can be partially accepted.

Clinically, there were no significant differences between the three conditioning modes of iBU in terms of “marginal staining” and “marginal adaptation”. At the same time, retention after 36 months was significantly lower in the ER mode than in the SE mode. In contrast, QMA showed the most marginal gap formation and the least perfect margins in the SE mode, while the ER and SEE modes showed higher marginal integrity. While marginal gap values were initially low in the iBU-ER group, they increased strongly at 36 months, consistent with increased restoration loss. This implies that prior dentin conditioning with phosphoric acid seems to harm the long-term bond stability of iBU. The universality claimed by the manufacturer could, therefore, not be achieved. Using optical coherence tomography, Merle et al. [29] showed that using iBU in ER mode resulted in more adhesive defects to dentin at 12 months than in SE mode. Accordingly, the loss of retention in the iBU-ER group started at this time. After 36 months, it was significantly higher than in the SE group. The iBU adhesive can be classified as a moderate adhesive system in terms of its pH value of 1.8 [14,61,62]. It is known that moderate one-step self-adhesive systems have a higher performance on dentin than strongly acidic systems in the SE mode [63] as the formation of water-insoluble monomer Ca salts is more pronounced [62]. Despite heterogeneous results depending on the adhesive used, in vitro results show that the initial bond strength of UAs in dentin does not differ significantly between SE and ER modes [3,11,64]. Regarding the long-term bond strength, contradictory results can be found, whereas the bonding in the SE mode tends to be more stable [5,64]. However, it should be noted that in vitro studies often use sound or freshly prepared dentin as a substrate, which has different bonding capacities than the sclerotic dentin found in NCCLs.

In a meta-analysis, Arbildo et al. [65] found that UAs in ER/SEE mode had better retention than those in SE mode. These results may indicate a particular effect of phosphoric acid-induced adhesion to enamel. Different application strategies were used on dentin for ER and SEE, with additional chemical adhesion more likely for SEE and SE. Therefore, one should be cautious in generalizing these results. Theoretically, we suspected an additive effect of microretentive and chemical adhesion when iBU was applied. It is unclear how pronounced the [62] adhesion of 10-MDP is in demineralized dentin. Possibly, phosphoric acid conditioning demineralizes sclerotic dentin less, leaving calcium binding sites for the functional monomer. However, the availability of calcium binding sites is expected to be reduced, potentially weakening chemical adhesion [3]. Thus, from the point of view of using a moderate acidic adhesive, the SE mode could be more beneficial. iBU performed best in the SEE mode, which is consistent with the current state of the literature: a recently published meta-analysis found better retention behavior for UAs in the SEE mode than in the SE mode [27]. In both this study and the meta-analysis, the universal adhesive was thus applied to the primarily relevant dentin surfaces in a self-conditioning manner, which confirms the previous assumption. Similarly, in the current study, the iBU-SEE group showed fewer marginal gaps and significantly more perfect marginal proportions than the iBU-SE group. SEE makes it possible to adapt well to the different substrates of the tooth structure. In dentin, the acidic monomers of the UAs demineralize only to a depth of 1 µm, which ensures complete infiltration of the monomer and stabilizes the collagen framework with the remaining hydroxylapatite [8]. Moreover, without phosphoric acid etching, enough calcium ions remain as binding sites to form stable salts with 10-MDP [3]. In enamel, on the other hand, the demineralization potential of the self-etching monomers is not strong enough to generate a sufficient microretentive pattern, and additional selective etching with phosphoric acid is recommended [5,27].

A strength of this study is that the fillings were placed by one experienced, calibrated dentist who was not involved in further clinical evaluation and QMA to minimize operator-related influences. To reduce the patient-related influence, the reference adhesive and the iBU were applied in three different modes to the same patient. Lesions were classified according to size, location, enamel, and dentin margins, but the degree of sclerosis was not determined. However, this seems to influence the adhesive bond [18]. In this regard, cavity preparation could be considered a limitation. It could only be somewhat standardized, but the influence was comparably distributed in all groups. In this study, QMA showed higher discriminatory power in two comparisons, and changes at the restoration margin could be detected early before they became clinically visible (iBU-SEE/OFL and iBU-ER/OFL). This was not the case in the system comparisons of iBU-SE/iBU-ER or iBU-SE/OFL. It is arguable that for the QMA in the groups iBU-ER and OFL, a total of one and four filling losses (Figure 1), respectively, were recorded, which may prevent statistical verification of a group difference, despite data imputation. On the other hand, the different assessment of the iBU-SE and iBU-ER groups with QMA clinically remains to be further investigated.

QMA using SEM provides a highly sensitive tool for morphological assessment, with gaps as small as 6 µm wide being displayable under the chosen imaging conditions (SEM and image analysis) and with the materials used for impression and replica fabrication. However, some method limitations impede a transfer of the results to the clinical situation: QMA can only evaluate the quality of the filling margins, as internal adhesive defects, which can cause a loss of retention, cannot be detected. Despite initial results confirming the continuation of marginal gaps into the cavity [37], it is not sufficiently known to what extent marginal integrity can be transferred to the bond quality of the entire interface. However, in a previous clinical study [66], it was shown that the QMA-based results on the integrity of the restoration margins were consistent with the assessment of tooth–composite bond failure at the entire tooth–composite interface and were in line with the clinical failure rates after three years. Restorations that exhibit poor marginal integrity may have sufficient bond strength in major parts of the internal interface [37]. In addition, marginal gaps can be masked by overhangs or biofilm [60]. In the SEM images of the present study, more bent-up restoration margins and partial fractures of the margin were observed before restoration loss. However, due to the definition of the evaluation criteria, these could often only be evaluated as a “positive ledge” and not as a “marginal gap”, even if a deficient adhesive bond could be assumed. In addition, the viscosity of the composite may have a relevant influence on the gaps. Various parameters of polymerization shrinkage or elasticity are potential influencing parameters. However, the literature is inconclusive and no clear trend can be verified (see [6,20] but also [67], thus further research is needed on this topic). Despite the strong influence of marginal quality, the clinical performance of an adhesive restoration is still multifactorial, making it challenging to define an acceptance threshold of marginal integrity that ensures clinical success [38]. QMA requires method-intensive scanning electron microscopy and is additionally time-consuming and material-intensive due to the preparation and evaluation effort. There are also various methodological limitations: impression taking for replica fabrication can lead to a loss of information, especially in the cervical and proximal regions [60]. The total restoration margin lengths and thus the measurable areas can vary in a longitudinal examination, and various impression inaccuracies may change the replica surface.

## 5. Conclusions

The application of iBU showed less retention loss than OFL at 36 months, and especially in the SEE and ER modes, iBU ensured the best marginal quality, with differences from the control appearing earlier with QMA than clinically. Thus, in restoring NCCls, iBU showed superior clinical performance compared to OFL, especially in SE and SEE modes. However, predictions for individual restorations are not possible, as even clinically acceptable restorations sometimes showed increased gap formation but were still in situ at 36 months.

## Figures and Tables

**Table 1:** Study groups and selection of teeth and lesions for clinical evaluation and quantitative margin analysis (QMA).

Group	iBU-SE	iBU-SEE	iBU-ER	OFL	Lost iBU-SE	Lost iBU-SEE	Lost iBU-ER	Lost OFL
N_clinic_/N_QMA_	50/11 ^1^	29/11 ^1^	50/11 ^1^	50/11 ^1^	1/1 ^2^	1/1 ^2^	8/7 ^2^	17/14 ^2^
Adhesive	iBond Universal			OptiBond FL	iBond Universal			OptiBond FL
Application Mode	self-etch (SE)	selective-enamel- etch (SEE)	etch-and-rinse (ER)	etch-and-rinse (ER)	self-etch (SE)	selective-enamel- etch (SEE)	etch-and-rinse (ER)	etch-and-rinse (ER)
Composite	Venus Diamond Flow							
Arc Distribution								
Maxillary	27/8	19/8	31/9	31/6	-/-	-/-	5/4	9/7
Mandibular	23/3	10/3	19/2	19/5	1/1	1/1	3/3	8/7
Tooth Distribution								
Incisor	8/1	8/2	12/2	11/2	-/-	-/-	1/1	4/2
Canine	12/2	6/2	14/5	12/3	1/1	-/-	2/2	2/2
Premolar	30/8	15/7	24/4	27/6	-/-	1/1	5/4	11/10
Lesion Borderline								
Enamel	-/-	-/-	-/-	-/-	-/-	-/-	-/-	-/-
Dentin	1/-	-/-	-/-	-/-	-/-	-/-	-/-	-/-
Mixed (enamel/dentin)	49/11	29/11	50/11	50/11	1//1	1/1	8/7	17/14
Lesion Depth								
Shallow (<1 mm)	6/-	9/3	8/1	6/-	-/-	1/1	1/1	2/2
Medium (1–2 mm)	42/11	19/8	41/10	42/11	1/1	-/-	7/6	13/12
Deep (>2 mm)	2/-	1/-	1/-	2/-	-/-	-/-	-/-	2/-

^1^ QMA: *n* = 11 patients recalled from BL to 36 m, randomly selected; ^2^ QMA: all restorations lost within the study period of the patients who appeared continuously from BL to 36 m.

**Table 2:** Composition of materials and their application according to the manufacturers’ recommendations.

Material		Composition	Application	Manufacturer
Etchant	Ultra-Etch^®^	35% H_3_PO_4_	iBU-SEE: Apply etchant on enamel for 30 s, rinse thoroughly for 15 s, air dry for 3 s (do not overdry) iBU-ER: Apply etchant on enamel for 30 s and on dentin for 15 s, rinse thoroughly for 15 s, air dry for 3 s (do not overdry) OFL: Apply etchant on enamel for 30 s and on dentin for 15 s, rinse thoroughly for 15 s, air dry for 3 s (do not overdry)	Ultradent Products, Inc.; South Jordan, UT, USA
Adhesive	iBond^®^ Universal	Methacrylate-monomer, 4-META, 10-MDP, acetone, water; pH 1.8 (LOT: 010021)	Active application of iBU for 20 s Carefully air dry with a gentle oil-free air flow until the adhesive film no longer moves, moving the air flow from outside to inside with increasing intensity, applying iBU again if lesion does not appear universally glossy. Light cure for 10 s (>1000 mW/cm^2^) ^1^	Kulzer GmbH; Hanau, Germany
	OptiBond^TM^ FL	Primer: HEMA, GPDM, MMEP, water, ethanol, photoinitiator (CQ), BHT (LOT: 5534310) Adhesive: Bis-GMA, HEMA, GPDM, GDMA, photoinitiator (CQ), ODMAB, fillers, barium aluminoborosilicate (LOT: 5592338)	Apply primer with brushing motion for 15 s Air dry for 5 s Apply adhesive with a light brushing motion for 15 s, air thin for 3 s Light cure for 20 s (>1000 mW/cm^2^) ^1^	Kerr GmbH; Herzogenrath, Germany
Composite	Venus^®^ Diamond Flow	UDMA, EBADMA, Ba-Al-F silicate glass, YbF_3_, SiO_2_, photoinitiators (LOT: 010113)	Apply in increments max. 2 mm, baseliner max. 1 mm Light cure each layer for 20 s (>1000 mW/cm^2^) ^1^	Kulzer GmbH; Hanau, Germany

10-MDP: methacryloyloxydecyl dihydrogen phosphate, 4-META: 4-methacryloyloxyethy trimellitate anhydride, BHT: butylhydroxytoluene, Bis-GMA: bisphenol A diglycidyl methacrylate, CQ: camphorquinone, EBADMA: ethoxylated bisphenol A dimethacrylate, GDMA: glycerol dimethacrylate, GPDM: glycerol phosphate dimethacrylate, HEMA: 2-hydroxyethyl methacrylate, ODMAB: 2-(ethylhexyl)-4-(dimethylamino)benzoate, UDMA: urethane dimethcarylate, ^1^ Regular curing light check with curing radiometer (Demetron Model 100, Demetron Res. Corp., Danbury, CT, USA).

**Table 3:** Clinical quality of the restorations from baseline (BL) up to 36 months (m); clinical data from a former study of BL up to 12 m [29].

	iBU-SE					iBU-SEE					iBU-ER					OFL				
	BL	6 m	12 m	24 m	36 m	BL	6 m	12 m	24 m	36 m	BL	6 m	12 m	24 m	36 m	BL	6 m	12 m	24 m	36 m
Restored teeth, n ^1^	50	49	47	45	45	29	28	26	24	23	50	49	47	45	44	50	49	45	39	33
Reassessment rate, %	100	98	94	90	90	100	96.6	89.7	82.8	79.3	100	98	94	90	88	100	98	90	78	66
Aesthetic criteria ^2^																				
Non-acceptable, %	0	0	0	0	0	0	0	0	0	0	0	0	0	0	2.6 ^3^	0	0	0	0	0
Functional criteria ^2^																				
Non-acceptable, %	0	0	0	0	2.2 ^4^	0	0	0	0	4.3 ^4^	0	0	2.1 ^4^	4.3 ^4^	17.4 ^4^	0	6.1 ^4^	16.7 ^4^	29.8 ^4^	36.2 ^4^
Biological criteria ^2^																				
Non-acceptable, %	0	0	0	0	0	0	0	0	0	0	0	0	0	0	0	0	0	0	0	0
Cumulative failure rate (total score) ^5^																				
Non-acceptable, %	0	0	0	0	2.2 ^4^	0	0	0	0	4.3 ^4^	0	0	2.1 ^4^	4.3 ^4^	19.6 ^6^	0	6.1 ^4^	16.7 ^4^	29.8 ^4^	36.2 ^4^

^1^ Total number of assessed restored teeth; ^2^ cumulative over time; ^3^ caused by subsurface staining; ^4^ caused by retention loss; ^5^ cumulative all criteria; ^6^ retention loss plus subsurface staining.

**Table 4:** Marginal staining, marginal adaptation (score 2 or 3, %), fractures/retention (score 5, %), and cumulative failure rate (total score). Group differences (p_i_) from 6 months up to 36 months (m); clinical data from a former study of 6 m up to 12 m [29]. For baseline, no group differences were calculable because all restorations were improved to score 1 before baseline rating.

	Time	%/p_i_	iBU-SE vs. iBU-SEE	iBU-SE vs. iBU-ER	iBU-SEE vs. iBU-ER	iBU-SE vs. OFL	iBU-SEE vs. OFL	iBU-ER vs. OFL
Marginal staining score 2 or 3	6 m	%	10.7/10.7	12.2/10.2	10.7/3.6	12.2/8.7	10.7/3.8	10.2/8.7
		p_i_	≥0.625					
	12 m	%	26.9/23.1	19.1/23.9	23.1/25.0	19.1/25.0	23.1/22.2	23.9/25.0
		p_i_	≥0.344					
	24 m	%	41.7/33.3	40.0/34.1	33.3/33.3	40.0/21.2	33.3/23.5	34.1/21.2
		p_i_	≥0.146					
	36 m	%	45.8/45.5	40.9/24.3	45.5/19.0	40.9/23.3	45.5/26.7	24.3/23.3
		p_i_	≥0.092					
Marginal adaptation score 2 or 3	6 m	%	50.0/46.4	40.8/53.1	46.4/53.6	40.8/50.0	46.4/46.2	53.1/50.0
		p_i_	≥0.286					
	12 m	%	50.0/46.2	53.2/53.2	46.2/44.0	53.2/70.0	46.2/68.2	53.2/70.0
		p_i_	1.000			0.064 ^1^	≥0.167	
	24 m	%	66.7/58.3	60.0/75.0	58.3/75.0	60.0/66.7	58.3/64.7	75.0/66.7
		p_i_	≥0.118					
	36 m	%	79.2/72.7	70.5/75.7	72.7/76.2	70.5/73.3	72.7/60.0	75.7/73.3
		p_i_	≥0.727					
Fractures and retention score 5 ^2^	6 m	%	0.0/0.0	0.0/0.0	0.0/0.0	0.0/6.1	0.0/7.1	0.0/6.1
		p_i_	n. c.			0.250	0.500	0.250
	12 m	%	0.0/0.0	0.0/2.1	0.0/3.8	0.0/16.7	0.0/18.5	2.1/16.7
		p_i_	n. c.	1.000	1.000	**0.016**	0.125	0.070 ^1^
	24 m	%	0.0/0.0	0.0/4.3	0.0/4.0	0.0/29.8	0.0/34.6	4.3/29.8
		p_i_	n. c.	1.000	1.000	**<0.001**	**0.016**	**0.003**
	36 m	%	0.0/4.3	2.2/17.4	4.3/12.5	2.2/36.2	4.3/42.3	17.4/36.2
		p_i_	1.000	0.070 **^1^**	1.000	**<0.001**	**0.016**	**0.039**
Cumulative failure rate (total score) ^3^	6 m	%	0.0/0.0	0.0/0.0	0.0/0.0	0.0/6.1	0.0/7.1	0.0/6.1
		p_i_	n. c.	n. c.	n. c.	0.250	0.500	0.250
	12 m	%	0.0/0.0	0.0/2.1	0.0/3.8	0.0/16.7	0.0/18.5	2.1/16.7
		p_i_	n. c.	1.000	1.000	**0.016**	0.125	0.070 ^1^
	24 m	%	0.0/0.0	0.0/4.3	0.0/4.0	0.0/29.8	0.0/34.6	4.3/29.8
		p_i_	n. c.	1.000	1.000	**<0.001**	**0.016**	**0.003**
	36 m	%	0.0/4.3	2.2/19.6	4.3/16.0	2.2/36.2	4.3/42.3	19.6/36.2
		p_i_	1.000	**0.039**	0.625	**<0.001**	**0.016**	0.146

Bold: significant; ^1^ trend; ^2^ retention loss, cumulative over time; ^3^ cumulative all criteria; n. c.: not calculable (McNemar, 2-sided); the percentages resulting from the group comparisons with the group iBU-SEE refer to *n* = 29.

**Table 5:** Changes (p_i_) in marginal staining, marginal adaptation (score 1 to 2 or 3), fractures/retention (score 1 to 5) and cumulative failure rate (all criteria) per group from baseline (BL) up to 36 months (m); clinical data from a former study of BL up to 12 m [29].

Parameter	Period	iBU-SE	iBU-SEE	iBU-ER	OFL
Marginal staining score 2 or 3	BL to 6 m	0.063 ^1^	0.250	0.125	0.250
	BL to 12 m	**0.021**	**0.031**	**0.002**	**0.004**
	BL to 24 m	**<0.001**	**0.008**	**<0.001**	**0.016**
	BL to 36 m	**<0.001**	**0.002**	**0.002**	**0.016**
Marginal adaptation score 2 or 3	BL to 6 m	**0.012**	0.109	**<0.001**	**0.001**
	BL to 12 m	**<0.001**	0.109	**<0.001**	**<0.001**
	BL to 24 m	**<0.001**	**0.039**	**<0.001**	**<0.001**
	BL to 36 m	**<0.001**	**0.001**	**<0.001**	**<0.001**
Fractures/retention score 5 ^2^ Cumulative failure rate (all criteria)	BL to 6 m	n. c.	n. c.	n. c.	0.250
	BL to 12 m	n. c.	n. c.	1.000	**0.016**
	BL to 24 m	n. c.	n. c.	1.000	**<0.001**
	BL to 36 m	1.000	1.000	**0.016^F/R^** **0.008^CFR^**	**<0.001**

Bold: significant; ^1^ trend; ^2^ retention loss, cumulative over time; n. c.: not calculable (McNemar, 2-sided).

**Table 6:** Means of marginal gap (%) and perfect margin (%) and group differences (p_i_) from baseline (BL) up to 36 months (m).

Parameter		iBU SE vs. SEE		iBU-SE vs. ER		iBU-SEE vs. ER		iBU-SE vs. OFL		iiBU-SEE vs. OFL		iBU-ER vs. OFL	
		%	p_i_	%	p_i_	%	p_i_	%	p_i_	%	p_i_	%	p_i_
Marginal gap	BL	1.5/0.0	0.125	1.5/0.2	0.125	0.0/0.2	0.250	1.5/2.5	0.426	0.0/2.5	**0.008**	0.2/2.5	**0.027**
	6 m	2.0/0.0	0.063 ^1^	2.0/0.9	0.438	0.0/0.9	0.063 ^1^	2.0/3.7	0.322	0.0/3.7	**0.004**	0.9/3.7	**0.012**
	12 m	4.3/0.5	0.078	4.3/1.8	0.313	0.5/1.8	0.063 ^1^	4.3/9.9	0.160	0.5/9.9	**0.004**	1.8/9.9	**0.020**
	24 m	6.8/1.4	**0.027**	6.8/3.7	0.164	1.4/3.7	0.219	6.8/8.7	0.232	1.4/8.7	**0.002**	3.7/8.7	**0.027**
	36 m	12.4/2.1	**0.014**	12.4/11.6	0.910	2.1/11.6	**0.016**	12.4/11.4	0.846	2.1/11.4	**0.002**	11.6/11.4	0.824
Perfect margin	BL	31.7/49.3	**0.024**	31.7/41.5	0.147	49.3/41.5	0.275	31.7/38.1	0.240	49.3/38.1	0.278	41.5/38.1	0.465
	6 m	20.3/39.7	**0.005**	20.3/32.9	0.067 ^1^	39.7/32.9	0.320	20.3/27.2	0.175	39.7/27.2	0.054 ^1^	32.9/27.2	0.278
	12 m	16.9/43.2	**0.001**	16.9/30.4	**0.042**	43.2/30.4	**0.027**	16.9/22.5	0.320	43.2/22.5	**0.003**	30.4/22.5	0.278
	24 m	11.8/30.7	**0.010**	11.8/22.6	**0.014**	30.7/22.6	0.275	11.8/11.3	0.695	30.7/11.3	**0.014**	22.6/11.3	**0.024**
	36 m	13.3/27.3	**0.032**	13.3/15.2	0.520	27.3/15.2	0.193	13.3/10.9	0.831	27.3/10.9	**0.014**	15.2/10.9	0.365

Bold: significant; ^1^ trend.

**Table 7:** Comparison of mean values of marginal gap (%) and perfect margin (%) of remaining restorations and those lost during the period.

Parameter		iBU-ER Remaining vs. Lost		OFL Remaining vs. Lost	
		%	p_i_	%	p_i_
Marginal gap, %	BL	0.1/0.5	0.581	3.4/2.6	0.494
	6 m	0.8/2.0	0.379	3.0/6.3	0.141
	12 m	1.7/2.8	0.612	5.2/9.8	0.394
	24 m	3.7/3.8	0.953	6.2/8.2	1.000
Perfect margin, %	BL	40.4/44.5	0.740	42.5/43.0	0.856
	6 m	30.6/31.4	1.000	25.3/32.7	0.056 ^1^
	12 m	29.2/23.3	0.536	27.8/25.0	0.694
	24 m	22.2/20.1	0.713	17.4/23.4	0.517

^1^ trend.

## Data Availability

Data supporting reported results are given in the respective tables.

## Supplementary Materials

The following supporting information can be downloaded at: https://www.mdpi.com/article/10.3390/jcm12185776/s1, Supplementary Table S1: Additional statistical informations.
