# Supplementary material for: Effect of the Adhesive Strategy on Clinical Performance and Marginal Integrity of a Universal Adhesive in Non-Carious Cervical Lesions in a Randomized 36-Month Study
Source: J Clin Med. 2023 Sep 5;12(18):5776. doi: 10.3390/jcm12185776 (PMC10531668; doi:10.3390/jcm12185776)
Supplement: Supplementary file 1 [file jcm-12-05776-s001.zip › jcm-2584351-supplementary.pdf]

Supplementary information

Supplementary Table S1 - Additional statistical information: Significant increases of mean values for marginal gap (Table 5) and significant decreases of perfect margin (p_i) within the groups from baseline (BL) up to 36 months (m).

Parameter	Period	iBU-SE	iBU-SEE	iBU-ER	OFL
Marginal gap	BL to 6 m	0.063[1]	1.000	0.063[1]	0.206
	BL to 12 m	0.078	0.500	0.063[1]	0.083
	BL to 24 m	**0.004**	0.125	0.063[1]	**0.019**
	BL to 36 m	**0.004**	0.063[1]	**0.016**	**0.019**
	6 m to 12 m	0.078	0.500	0.063[1]	**0.004**
	6 m to 24 m	**0.004**	0.125	0.063[1]	**0.002**
	6 m to 36 m	**0.004**	0.063[1]	**0.016**	**0.002**
	12 m to 24 m	**0.004**	0.125	0.063[1]	0.416
	12 m to 36 m	**0.004**	0.063[1]	**0.016**	0.311
	24 m to 36 m	**0.004**	0.063[1]	**0.016**	**0.002**
Perfect margin	BL to 6 m	**0.032**	**0.024**	**0.019**	0.147
	BL to 12 m	**0.001**	0.278	**0.005**	**0.042**
	BL to 24 m	**0.007**	**0.032**	**0.001**	**0.002**
	BL to 36 m	**0.019**	**0.006**	**0.001**	**0.001**
	6 m to 12 m	0.278	0.320	0.520	0.520
	6 m to 24 m	**0.042**	0.260	**0.019**	**0.019**
	6 m to 36 m	0.413	**0.019**	**0.014**	**0.014**
	12 m to 24 m	0.084	0.083	**0.032**	**0.005**
	12 m to 36 m	0.413	**0.019**	**0.005**	**0.001**
	24 m to 36 m	0.320	0.240	**0.007**	0.830

Bold: significant; [1]trend.
